# Predicting chemotherapy response in non-small-cell lung cancer *via* computed tomography radiomic features: Peritumoral, intratumoral, or combined?

**DOI:** 10.3389/fonc.2022.915835

**Published:** 2022-08-08

**Authors:** Runsheng Chang, Shouliang Qi, Yifan Zuo, Yong Yue, Xiaoye Zhang, Yubao Guan, Wei Qian

**Affiliations:** ^1^ College of Medicine and Biological Information Engineering, Northeastern University, Shenyang, China; ^2^ Key Laboratory of Intelligent Computing in Medical Image, Ministry of Education, Northeastern University, Shenyang, China; ^3^ Department of Radiology, Shengjing Hospital of China Medical University, Shenyang, China; ^4^ Department of Oncology, Shengjing Hospital of China Medical University, Shenyang, China; ^5^ Department of Radiology, The Fifth Affiliated Hospital of Guangzhou Medical University, Guangzhou, China

**Keywords:** non-small cell lung cancer, Computed Tomography (CT), chemotherapy response, radiomics, peritumoral features, area under curve

## Abstract

**Purpose:**

This study aims to evaluate the ability of peritumoral, intratumoral, or combined computed tomography (CT) radiomic features to predict chemotherapy response in non-small cell lung cancer (NSCLC).

**Methods:**

After excluding subjects with incomplete data or other types of treatments, 272 (Dataset 1) and 43 (Dataset 2, external validation) NSCLC patients who were only treated with chemotherapy as the first-line treatment were enrolled between 2015 and 2019. All patients were divided into response and nonresponse based on the response evaluation criteria in solid tumors, version 1.1. By using 3D slicer and morphological operations in python, the intra- and peritumoral regions of lung tumors were segmented from pre-treatment CT images (unenhanced) and confirmed by two experienced radiologists. Then radiomic features (the first order, texture, shape, et al.) were extracted from the above regions of interest. The models were trained and tested in Dataset 1 and further validated in Dataset 2. The performance of models was compared using the area under curve (AUC), confusion matrix, accuracy, precision, recall, and F1-score.

**Results:**

The radiomic model using features from the peritumoral region of 0–3 mm outperformed that using features from 3–6, 6–9, 9–12 mm peritumoral region, and intratumoral region (AUC: 0.95 versus 0.87, 0.86, 0.85, and 0.88). By the fusion of features from 0–3 and 3–6 mm peritumoral regions, the logistic regression model achieved the best performance, with an AUC of 0.97. This model achieved an AUC of 0.85 in the external cohort. Moreover, among the 20 selected features, seven features differed significantly between the two groups (p < 0.05).

**Conclusions:**

CT radiomic features from both the peri- and intratumoral regions can predict chemotherapy response in NSCLC using machine learning models. Combined features from two peritumoral regions yielded better predictions.

## Introduction

Lung cancer remains the leading cause of cancer-related deaths, with a 2-year relative survival rate of 36% (1). Histologically, non-small cell lung cancer (NSCLC) is the most common type of lung cancer, and locally advanced NSCLC patients comprise approximately 30% of newly diagnosed patients (2–4). Clinically, patients received surgery, chemotherapy, radiation, or targeted drug therapies as the first-line treatment according to related clinical guidelines. As the standard first-line treatment of advanced-stage NSCLC patients with no specific gene mutations, chemotherapy has been and will still be a cornerstone in the near future (5). However, owing to the heterogeneity of tumors, different patients may have extremely different therapeutic effects on chemotherapy, and the adverse reaction may even have a significant impact on the survival rate of NSCLC patients (6–10).

Radiomic features, extracted from computed tomography (CT) images, can quantitatively express crucial information regarding the physiology of the entire tumor, including the intra-tumor and its surroundings (11–13). Owing to the spatially and temporally heterogeneous nature of tumors, these features can quantify the phenotypic differences from a high-dimensional space that cannot be distinguished by the naked eye. Therefore, these features and the resulting radiomic models are of important guiding significance for precision oncology and can improve decision support in prognosis and therapeutic response prediction at a low cost (14, 15).

Recently, many studies have begun investigating the role of radiomics features of the surrounding area of the lesion (peritumoral region) in disease screening, prediction of treatment response, and prognosis. The microenvironment and habitat surrounding the tumor may play an extremely important role in predicting prognosis. Many studies have found that the pathogenesis and progression of lung cancer are closely related to tumor-infiltrating lymphocytes and tumor-associated macrophages all over the tumor microenvironment (Maeda et al) (16–18). Algohary *et al.* studied 231 prostate cancer patients and extracted radiomic features from the intra- and peri-tumoral region of interest (ROI) to distinguish prostate cancer risk categories as defined by the D’Amico Risk Classification System, with an area under the receiver operating characteristic curve (AUC) of 0.84 (19). Shan *et al.* constructed a model based on peritumoral radiomic signatures from CT images of 156 patients to predict the early recurrence of hepatocellular carcinoma after curative treatment and obtained an AUC of 0.80 (20).

Many radiomics studies have also been applied to the treatment of NSCLC. Khorrami *et al.* collected 125 NSCLC patients to identify the role of radiomics texture features from regions both within and outside the nodule in predicting response to chemotherapy and overall survival; they obtained an AUC of 0.82 (21). Braman *et al.* analyzed intra- and peritumoral regions of 117 patients with breast cancer to predict pathological complete response to neoadjuvant chemotherapy and obtained an AUC of 0.78 (22).

However, the ability of peritumoral, intratumoral, or combined CT radiomic features to predict chemotherapy response in NSCLC has not been well studied. In this study, we established different CT radiomic models using features from different peritumoral, intratumoral, or combined regions and evaluated their performance in predicting chemotherapy response in NSCLC.

## Materials and methods

### Patient characteristics

This study was approved by the ethics committee of Shengjing Hospital of China Medical University and the Fifth Affiliated Hospital of Guangzhou Medical University, and the requirement for informed consent was waived because this was a retrospective study. A total of 605 patients with NSCLC were enrolled between 2015 and 2019 at Shengjing Hospital of China Medical University. Of these 605 patients, 272 NSCLC patients who were treated with chemotherapy alone as first-line treatment were included in this study (Dataset 1). **Supplemental Figure S1** shows the two steps of the exclusion criteria. Using the same criteria, 43 patients from the Fifth Affiliated Hospital of Guangzhou Medical University were selected and used as the external validation cohort (Dataset 2).

The clinical characteristics of the patients are presented in **Table 1**. Pathologic stage was characterized according to the seventh edition of the American Joint Committee on Cancer TNM staging system. For each patient, non-contrast CT images were acquired before and after chemotherapy. The parameters used for CT image acquisition are listed in **Supplemental Table S1**.

**Table 1:** Clinical characteristics of NSCLC patients.

		Dataset 1			Dataset 2		
Characteristics		Response group	Non response group	*p*-value	Response group	Non response group	*p-*value
No. of patients		148	124	–	24	19	–
Gender	Male	81	69	3.843^a^	22	15	4.987^a^
	Female	67	55		2	4	
Age, median (SD), y		63.76 (11.30)	64.86 (10.65)	0.453^b^	66.42 (9.86)	62.36 (14.58)	0.629^b^
Smoking status	Ever	50	69	1.021^a^	17	12	1.235^a^
	Never	98	55		7	7	
Histological type	Adenocarcinoma	121	101	2.241^a^	13	11	3.244^a^
	Squamous cell carcinoma	27	23		11	8	
TNM Stage	II	22	16	1.232^a^	1	2	2.065^a^
	III	118	103	0.863^a^	20	15	0.983^a^
	IV	8	5	1.528^a^	3	2	1.024^a^
Courses, median (SD)		4.56 ± 1.41	3.87 ± 2.04	0.002^b^	3.87± 1.58	3.24± 1.06	0.688^a^

^a^p value of Chi-square test; ^b^p value of two-sample t-test.

SD, standard deviation; TNM, tumor node metastasis classification.

According to the response evaluation criteria in solid tumors (RECIST, version 1.1) (23), clinical responses were categorized into four parts by comparing CT images collected before and after chemotherapy: (I) complete response (CR): all target lesions disappeared; (II) partial response (PR): the target lesions decreased by at least 30% in the sum of the diameters; (III) progressive disease (PD): the target lesions increased by at least 20% in the sum of the diameters; (IV) stable disease (SD): neither sufficient shrinkage to qualify for PR nor sufficient increase to qualify for PD. The interval between CT scans before and after chemotherapy was 4.56 ± 1.41 and 3.87 ± 2.04 treatment courses in response and nonresponse groups (each treatment course takes three weeks) of Dataset 1. The interval was 3.87± 1.58 and 3.24± 1.06 treatment courses in the two groups of Dataset 2.

In this study, clinical response was defined as “response” and “nonresponse” based on the radiologist’s evaluation *via* RECIST and clinical manifestations. The response group included patients with CR and PR, while the non-response group included patients with PD and SD.

### Overview of the study procedure

**Figure 1:**
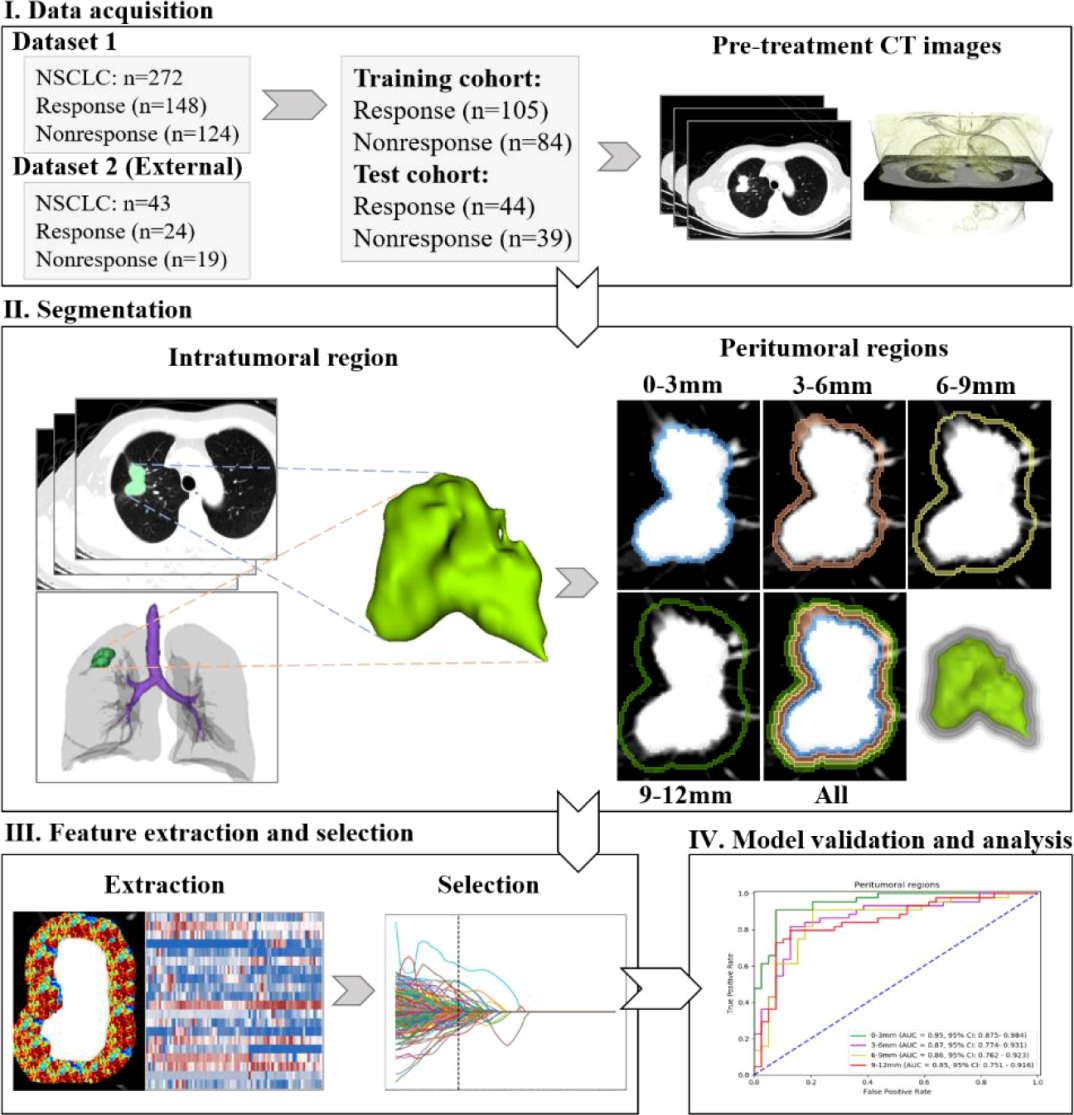
Overview of the whole study procedure.

**Figure 1** shows a brief procedure of this study. First, the 272 NSCLC patients (148 responses and 124 nonresponses) were randomly divided into a training cohort of 189 patients (105 responses and 84 nonresponses) and an independent test cohort of 83 patients (44 responses and 39 nonresponses). Second, all lesions were segmented from the pre-treatment CT images, and then the peritumoral regions (0–3 mm, 3–6 mm, 6–9 mm, and 9–12 mm) around the lesion. Third, radiomics features were extracted from the segmented regions, and discriminative features were selected. Finally, different models were trained using radiomic features, validated, and compared.

### Segmentation of intra- and peritumoral regions

First, to eliminate interference factors, all pre-treatment CT images of the NSCLC patients were interpolated into voxels of 1×1×1 mm. Thereafter, intratumoral regions were semi-automatically segmented from these CT images by two radiologists with more than 15 years of experience using 3D Slicer software (24). By adding seed points and applying the fast marching method, the lesions could be quickly segmented automatically. If necessary, the errors were corrected by radiologists manually. To compare the segmentation by the two radiologists, the Dice coefficient and over– and under–lesion segmentation errors were calculated.

Next, four morphological dilation operations were applied with the number of pixels of 3, 6, 9, and 12, respectively. These operations were based on a 3D morphology algorithm in the skimage package (https://scikit-image.org). After subtraction, four peritumoral regions of 0–3 mm, 3–6 mm, 6–9 mm, and 9–12 mm were obtained. **Supplemental Figure S2** shows the details of these regions.

### Radiomic features extraction and selection

First, an open-source PyRadiomics Python package was applied to extract 1688 radiomic features from each segmented region. To establish a reference standard for radiomics analysis, PyRadiomics provides an open-source platform for easy and reproducible radiomic feature extraction (25). The original CT images and derived 19 categories of images (LoG with five sigma levels, one level of wavelet decompositions yielding eight derived images and images derived using square, square root, logarithm, exponential, gradient, and local binary pattern filters) were utilized to extract the features. 1896 radiomics features including the first order (380), shape-based (16), gray-level co-occurrence matrix (480), gray level run length matrix (320), gray level size zone matrix (320), neighboring gray tone difference matrix (100), and gray level dependence matrix (280) were obtained. After removing the unusable ones, 1688 features were retained.

Next, for each intra- or peritumoral region, 20 discriminative radiomics features were selected using the least absolute shrinkage and selection operator (LASSO) algorithm. The LASSO algorithm adds a penalty term (λ) to the loss function (optimization target); therefore, λ is considered in the process of training and solving parameters. As shown in **Supplemental Figure S3**, with an increase in λ, the mean square error decreases gradually to the lowest point. This point corresponds to the optimal parameter of λ. Meanwhile, the coefficient of the less influential feature will decrease to 0, and finally, only the most important features are retained (26). At the optimal λ, features with non-zero coefficients will be retained and ranked by the absolute value of the coefficient. To decrease the overfitting risk and avoid the dimensionality curse, only the top 20 features are finally selected as the discriminative features according to the rule of thumb that each feature corresponds to 10 samples in a binary classifier (27).

### Model construction, validation, and comparison

To clarify the performance of models using features from different peri- and intratumoral regions, four groups of comparative experiments were conducted.

- To investigate features from which peritumoral regions perform best, the four models corresponding to 0–3 mm, 3–6 mm, 6–9 mm, and 9–12 mm are compared.
- To investigate whether the fusion of peritumoral features and images improves the performance, models using the feature and image fusion of 0–3 mm and 3–6 mm were compared (28–30).
- To consider whether peritumoral features outperform intratumoral features, a model using features from the intratumoral region was studied.
- To explore whether the fusion of peri- and intratumoral features and images improves the performance, the models using the feature and image fusion of intratumoral and 0–3 mm peritumoral regions were compared.

Feature fusion implies that 1688 features from each region are combined into 3376 features, and the top 20 features are selected according to the same method described previously. Image fusion implies that the two regions are combined, 1688 features are extracted, and the top 20 are maintained in the same way.

Different models were constructed using three representative machine-learning classifiers: random forest (RF), support vector machine (SVM), and logistic regression (LR). Each optimal hyper-parameter of the models was calculated using a grid search algorithm and 10-fold cross-validation. This implies that every grid of hyper-parameters is evaluated by the average of 10-fold cross-validation, and a combination of optimal hyper-parameters is obtained after traversing all grids. The model with optimal hyper-parameters was retrained using all training data (n=189), and then the generated model was evaluated in an independent test cohort (n=83). The aim of dividing Dataset 1 into a training cohort and a test cohort is to obtain the optimal hyper-parameters in machine-learning classifiers and simultaneously avoid information leakage. Dataset 2 was used as an external validation cohort to know the generalizability of the model developed in Dataset 2. The two datasets were collected from different hospitals and by different CT scanners.

Specifically, we used a grid search with cross-validation (GridSearchCV) to traverse the hyper-parameters within a certain range and with a specific interval. In SVM, the kernel parameter was set as “linear” or radial basis function (“rbf”); the parameter C was set as 0.001, 0.01, 0.1, 1, 10, 100 or 1000; the gamma parameter was set as 0.0001, 0.001, 0.005, 0.01, 0.1, 0.5, 1, 3, 5, 10 or 100. In RF, n_estimators parameter ranged from 20 to 2000 with an interval of 10, max_features parameter was set as 2 or 3, min_sample_leaf ranged from 1 to 50 with an interval of 1 and ranged from 100 to 500 with an interval of 50. In LR, the C parameter was set as 0.001, 0.01, 0.1, 1, 10, or 100; the penalty item was set as L1 or L2.

### Model evaluation and statistical analysis

For each model, the performance was evaluated by the area under the receiver operating characteristic curve (AUC) with 95% confidence interval (CI), confusion matrix, accuracy, precision, recall, and F1-score. The cut-off was determined using Youden’s index and the shortest distance from the coordinate (0, 1) on the ROC curve.

A two-sample t-test was used to compare the age and number of treatment courses between the response and non-response groups. The chi-square test was used to compare the gender, histological type, and smoking status of the two groups. The ROC curves of the different models were compared using the Delong test. If p<0.05, a significant difference was considered to be statistically significant.

## Results

### Performance of features from different peritumoral regions

**Figure 2:**
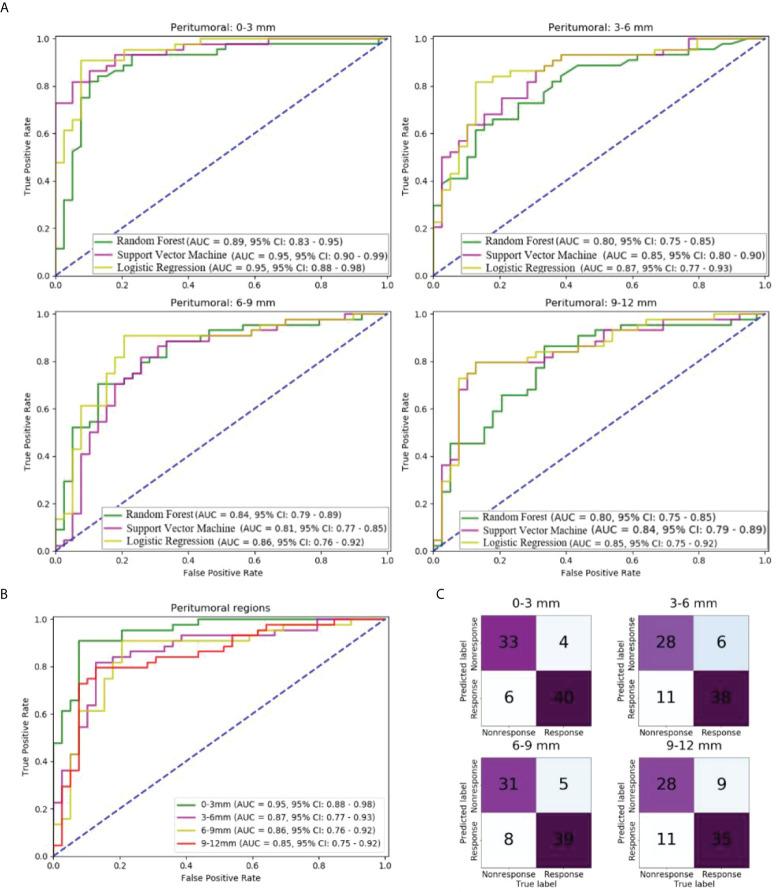
Comparison of models using different peritumoral regions in the independent test cohort: **(A)** ROC curves of models using different peritumoral regions and machine learning methods; **(B)** ROC curve of models using different peritumoral regions and logistic regression; **(C)** Confusion matrix of models using different peritumoral regions and logistic regression.

In the independent test cohort, the predictive performance of the three machine-learning models in each peritumoral region is shown in **Figure 2**. It was found that among the three machine learning classifiers, the LR model presented the highest AUC and performed best in every peritumoral region (**Figure 2A**). The ROC curve and confusion matrix of LR models using features from four peritumoral regions are summarized in **Figures 2B, C**, respectively. The AUC of 0–3, 3–6, 6–9, and 9–12 mm peritumoral regions were 0.95, 0.87, 0.86, and 0.85, respectively. The peritumoral region of 0–3 mm had the highest AUC.

The other performance measures are listed in **Table 2**. For the 0–3 mm peritumoral region, the accuracy, precision, recall, and F1-score were 87.9%, 0.89, 0.85, and 0.87, respectively, while the cut-off value was 0.83. For the 3–6 mm peritumoral region, the measures were 79.5%, 0.82, 0.72, and 0.77, while the cut-off value was 0.69. For the 6–9 mm peritumoral region, the measures were 84.3%, 0.86, 0.80, and 0.83, while the cut-off value was 0.70. For the 9–12 mm peritumoral region, the measures were 75.9%, 0.76, 0.72, and 0.74, respectively, while the cut-off value was 0.67.

**Table 2:** Predictive performance of different regions in the independent test cohort.

ROI	AUC	Accuracy	Precision	Recall	F-score
0-3 mm	0.95	87.9%	0.89	0.85	0.87
3-6 mm	0.87	79.5%	0.82	0.72	0.77
6-9 mm	0.86	84.3%	0.86	0.80	0.83
9-12 mm	0.85	75.9%	0.76	0.72	0.74
Image fusion (0–3 and 3–6 mm)	0.89	80.7%	0.85	0.72	0.78
Feature fusion (0–3 and 3–6 mm)	0.97	92.7%	0.922	0.92	0.92
Intratumoral region	0.88	81.9%	0.85	0.74	0.80
Image fusion (Intra and 0–3 mm)	0.88	81.9%	0.82	0.80	0.81
Feature fusion (Intra and 0–3 mm)	0.92	91.5%	0.94	0.87	0.91

ROI, region of interest; AUC, area under the curve.

### Performance of different methods of fusing peritumoral regions

**Figure 3:**
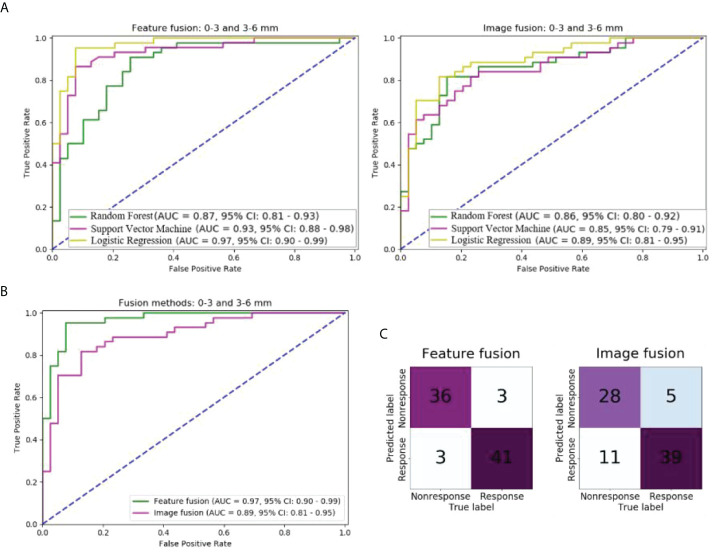
Comparison of models with different fusion methods of 0–3 and 3–6 mm peritumoral regions in the independent test cohort: **(A)** ROC curves of models of two fusion methods and three machine learning methods; **(B)** ROC curves of models of two fusion methods and logistic regression; **(C)** Confusion matrix of models of two fusion methods and logistic regression.

In the independent test cohort, the predictive performance of models using the feature and image fusion of 0–3 mm and 3–6 mm peritumoral regions were compared (**Figure 3**). As shown in **Figure 3A**, the LR model outperformed the SVM and RF models in both feature fusion and image fusion. For the LR model, the feature fusion and image fusion were compared using the ROC curve and confusion matrix (**Figures 3B, C** **)**. The AUC of feature fusion of 0–3 and 3–6 mm peritumoral regions was 0.97, higher than that of image fusion (AUC of 0.89). The LR model using feature fusion of 0–3 and 3–6 mm peritumoral regions can correctly predict 36 of 39 nonresponse patients and 41 of 44 response patients.

The other performance measures of these two models are listed in **Table 2**. The model of feature fusion achieved an accuracy of 92.7%, precision of 0.92, recall of 0.92, an F1-score of 0.92, and a cut-off value of 0.88. For the image fusion model, the four measures were 80.7%, 0.85, 0.72, and 0.78, respectively, while the cut-off value was 0.69.

### Performance of intratumoral region

The ROC curve and confusion matrix of models using CT radiomic features from the intratumoral region are shown in **Supplemental Figure S4**. Among the three models, the LR model performed the best, with an AUC of 0.88. In the independent test cohort, 29 of 39 non-response patients and 39 of 44 response patients were correctly predicted by the LR model. The cut-off value was 0.71, and the accuracy, precision, recall, and F-score were 81.9%, 0.85, 0.74, and 0.80, respectively (**Table 2**).

### Performance of different methods of fusing intra and peritumoral regions

**Supplemental Figure S5** shows the performance of radiomic models using different methods of fusing intra and 0–3 mm peritumoral regions. Similar to the previous results, the LR model outperformed the SVM and RF models for both fusion methods (image and feature) (**Supplemental Figure S5A**); the AUC was 0.88 for the LR model using the image fusion method and it was 0.92 using the feature fusion (**Supplemental Figures S5B, C**). Feature fusion yields better performance than image fusion. For the LR model using the image fusion method, the accuracy, precision, recall, and F-score were 81.9%, 0.82, 0.80, 0.81, and 0.67, respectively, while the cut-off value was 0.83. For the LR model using the image method, it was 91.5%, 0.94, 0.87, and 0.91, while the cut-off value was 0.83.

**Figure 4:**
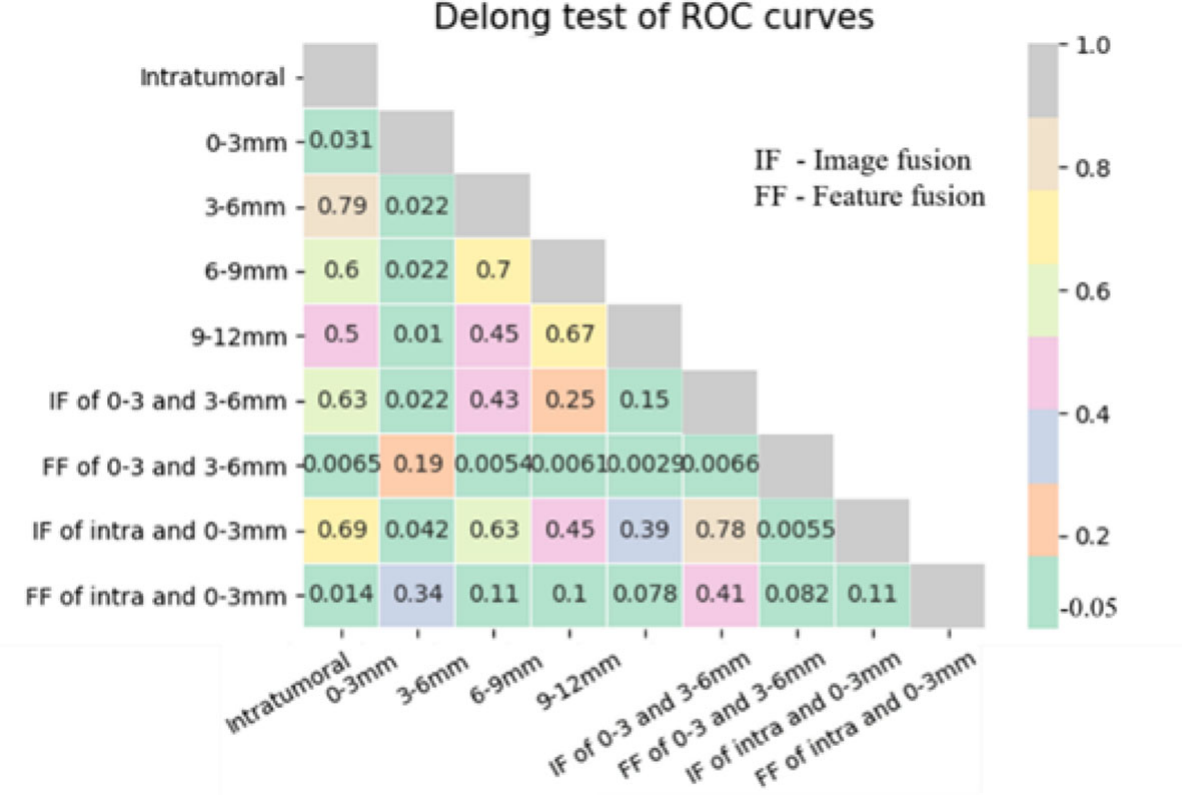
p values of Delong test between ROC curves of different models.

The p values in the Delong test of ROC curves of nine different models are shown in **Figure 4**. The AUC of the LR model using the 0–3 mm peritumoral region was significantly higher than that of the three models using the 3–6, 6–9, and 9–12 mm peritumoral regions and that of the model using the intratumoral region (Delong test, p<0.05). Feature fusion of 0–3 and 3–6 mm peritumoral regions produced an AUC significantly higher than that in the six cases of 3–6, 6–9, and 9–12 mm peritumoral regions, intratumoral regions, image fusion of 0–3 and 3–6 mm peritumoral regions, and image fusion of intratumoral and 0–3 mm peritumoral regions (Delong test, p<0.05). Although the AUC of the model using feature fusion of 0–3 and 3–6 mm peritumoral regions was higher than that of the other two cases of 0–3 mm peritumoral region and feature fusion of intratumoral and 0–3 mm peritumoral regions, no significant difference was observed (Delong test, p>0.05).

### Radiomic features over traditional clinical features

**Supplemental Figure S6** shows the performance of models with radiomics features and clinical features (gender, age, histological type, TNM stage, smoking status and the number of treatment courses). The AUC of the model with only clinical features was 0.55. While using both radiomics and clinical features, the model achieved an AUC of 0.96, even lower than that only using radiomic features (0.97). It demonstrates that clinical features had no improvement in predicting chemotherapy in this research.

### Performance in the external validation dataset

**Figure 5:**
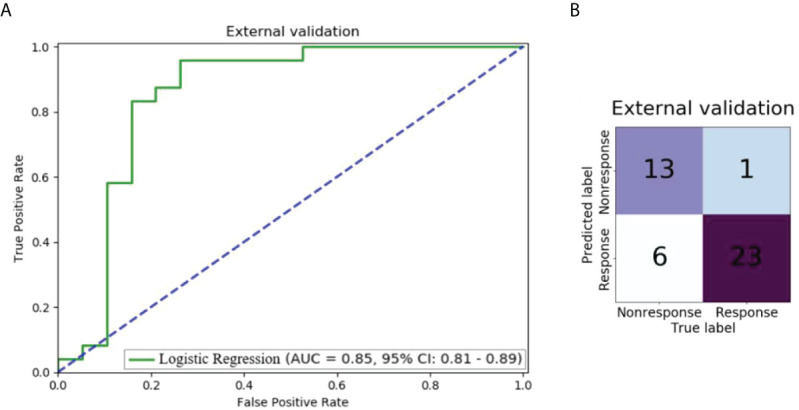
Performance of the model using 0-3 and 3-6 peri-tumoral features in the external validation dataset: **(A)** ROC curve; **(B)** Confusion matrix.

**Figure 5** shows the performance of the model using 0-3 and 3-6 peri-tumoral features in the external validation dataset. The AUC was 0.85 (95% CI: 0.81-0.89) and 13 of 19 non-response patients and 23 of 24 response patients were correctly predicted by the LR model.

### Segmentation agreement and characteristics of radiomic features

For the segmentation agreement by two radiologists, the Dice coefficient is 0.85 ± 0.06, and the over- and under-segmentation errors of segmented tumor volume are 0.22 ± 0.14, 0.28 ± 0.03, respectively.

**Figure 6:**
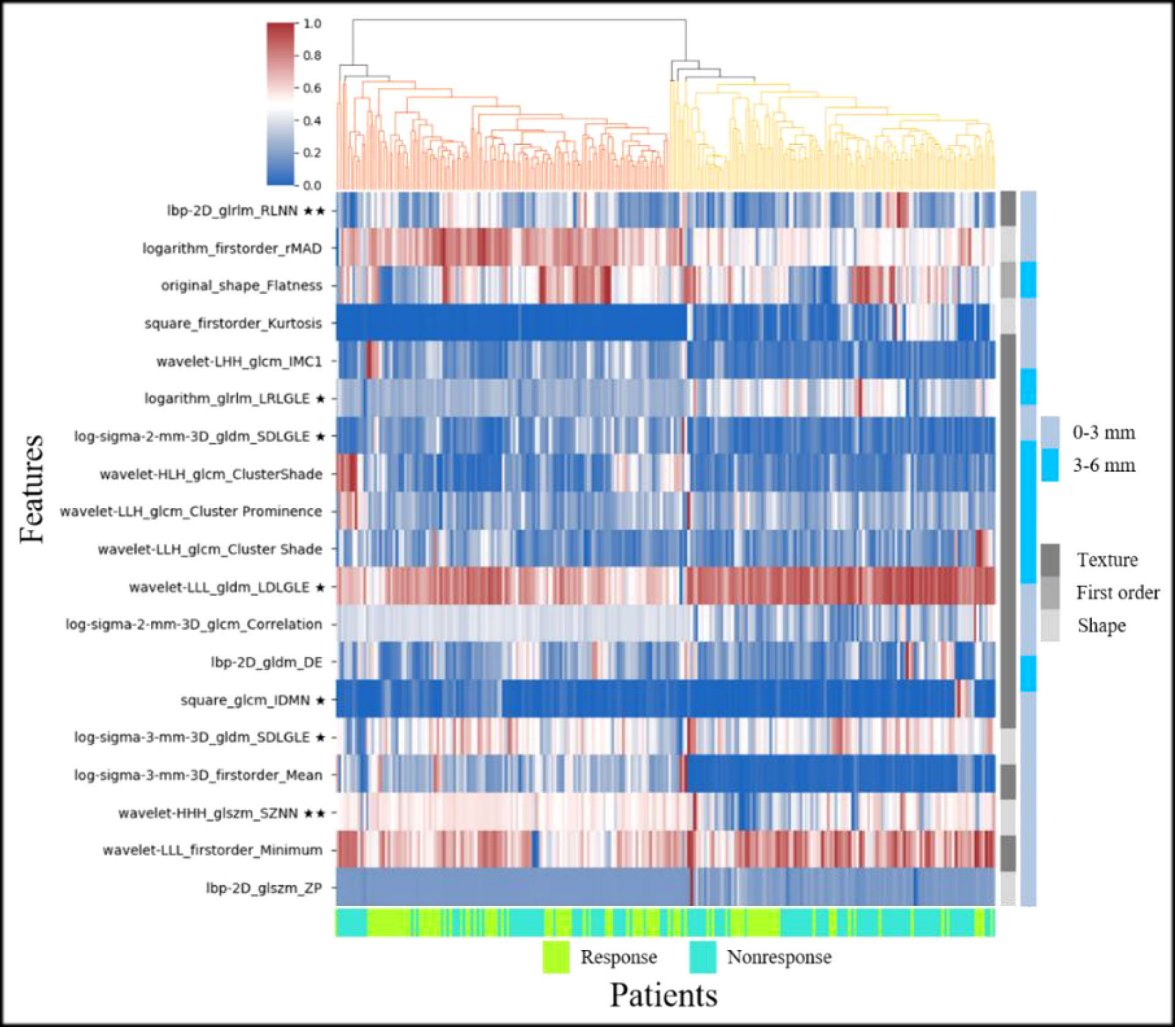
Heat map and dendrogram of the top 20 radiomic features in response and nonresponse groups of the training set (★ indicates p < 0.05, ★★ indicates p < 0.001).

Feature fusion of 0–3 and 3–6 mm peritumoral regions had the highest AUC in all nine cases. In this case, the 20 discriminative radiomic features (13 from 0–3 mm, 7 from 3–6 mm) included five first-order features, one shape feature, and 14 texture features. Seven radiomic features were significantly different between the response and nonresponse groups [two features with p<0.001(★★) and five features with p<0.05(★)]. **Figure 6** shows the unsupervised hierarchal clustering of radiomic features in the training set, where the x-axis represents the training cohort of patients (n = 189) and the y-axis represents the 20 radiomic features.

## Discussions

In this study, the ability of peritumoral, intratumoral, or combined CT radiomic features to predict chemotherapy response in NSCLC was evaluated. It was found that the radiomic model using features from 0–3 mm peritumoral region outperforms that using features from 3–6 mm, 6–9 mm, 9–12 mm peritumoral region, and intratumoral region, with the highest AUC of 0.95. By fusing features from 0–3 mm and 3–6 mm peritumoral regions, the AUC can be further 0.97. Two over-represented features in the response group indicated higher heterogeneity of NSCLC tumors.

### Is the peritumoral region predictive?

Our results demonstrated that CT radiomic features from peritumor regions are predictive of chemotherapy response in NSCLC. The prognosis of lung cancer is not only reflected in the lesion but also the surrounding normal tissues; thus, the microbial environment also has great predictive potential for the response to clinical treatment (31). The microenvironment of the peritumoral region of breast malignancy is related to aggressiveness (22). The capillaries and various cells around the tumor border might be more active than those inside the tumor; thus, their immune response to cancer and response to the prognosis, such as chemotherapy, is probably more severe. Algohary *et al.* studied the density of stromal macrophages, epithelial cells, and lymphocytes in the peritumoral region and found it to be related to metastasis of prostate cancer risk.^19^ Matsumura et al. collected 1069 resected NSCLC patients with lymphatic permeation located in intra-, peritumoral, or absent to determine the survival impact, and found that lymphatic canals present in peritumoral regions have a significantly higher overall survival rate than the other two groups (32).

### Which peritumoral region is optimal?

Similar to the recommended negative surgical margin in the clinic, the different ranges of the peritumoral region contribute significantly to the prediction of prognostic response. We have found that the features from the 0–3 mm peritumoral region are more predictive of the chemotherapy response of NSCLC than those from 3–6 mm, 6–9 mm, and 9–12 mm peritumoral regions. Some previous studies have indicated that the region beyond 15 mm around the lung tumor lesion has no contribution to predicting the recurrence or remission (21, 33). Beig *et al.* showed that low and middle frequencies of Gabor filters had a higher response at 5 mm around the adenocarcinomas lesion (23). Braman *et al.* found that features from the 2.5–5.0 mm region surrounding the breast tumor are predictive of the pathological complete response to neoadjuvant chemotherapy (22). Algohary *et al.* have found that Haralick from 3–6 and 6–9 mm peritumoral rings and CoLlAGe texture features from 6–9 mm ring were over- and under-expressed, respectively, in high-risk prostate cancer lesions (19).

### Is the peritumoral region superior to the intratumoral region?

Our study has shown that the peritumoral region is superior to the intratumoral region in predicting chemotherapy response in NSCLC. A growing number of studies have proven that the tissues and microenvironment around the tumor can provide unique effects on radiomic analysis, sometimes exceeding the intratumoral region (34). Braman *et al.* analyzed the tumor and its surroundings of breast cancer and found that the peritumoral region performed better in estimating the response to HER2-targeted neoadjuvant therapy (35).

### Does the combination of regions improve prediction?

In this study, we investigated models using different methods of fusing two peritumoral regions. The model using feature fusion of 0–3 and 3–6 mm peritumoral regions achieves an AUC of 0.97, which is higher than that of the model using the 0–3 mm peritumoral region (0.95), although there was no significant difference (Delong test, p=0.19). The feature fusion of the 0–3 mm peritumoral region and intratumoral region even decrease the AUC from 0.95 (only using features from 0–3 peritumoral region) to 0.92. However, Jiang *et al.* have reported that a combination of intra- and peritumoral features of gastric cancer can improve the prediction of chemotherapy response (36). Chen *et al.* also found that incorporating peritumoral radiomic analysis of hepatocellular cancer with intratumoral features can improve the immunoscore estimation of hepatocellular cancer (37). Hu *et al.* have shown that the combination of intra- and peritumoral features can improve the performance in estimating pathological complete response after neoadjuvant chemoradiation in patients with oesophagal squamous cell carcinoma (38). Therefore, we thought that whether the combination of regions improves prediction might depend on two aspects: discriminative and supplementary. If the features from different regions are both discriminative and supplementary, the combination will improve the prediction. Otherwise, the results of the combination are uncertain.

Moreover, we found that feature fusion was better than image fusion for prediction. This might be because each feature extraction method might have an upper limit of capability. After the combination of images from different regions, the 1688 extracted features are representative of the entire region. However, the feature fusion method combines features extracted from two regions into a set of 3376 features and then uses feature selection methods to obtain the discriminative features. Therefore, complementary features from two different regions can remain. This might be the reason why most previous studies have adopted feature fusion methods (36–38).

### Does higher heterogeneity in the peritumoral region correspond to response?

In the response group, run length non-uniformity normalized (RLNN) and size zone nonuniformity normalized (SZNN) features were overrepresented (i.e., higher than that in the nonresponse group). The RLNN measures the similarity of run lengths throughout the image, with a lower value indicating greater homogeneity among run lengths in the image. SZNN measures the variability of size zone volumes throughout the image, with a lower value indicating greater homogeneity among the zone size volumes in the image.

One constructive finding of this research is that in the peritumoral region of NSCLC lesions, the response group had higher heterogeneity than the nonresponse group. Specifically, SZNN and RLNN were overrepresented. This finding provides further evidence that the heterogeneity of the microenvironment in both the tumor and the area around the tumor is predictive of the prognosis of lung cancer. This heterogeneity might be reflective of genomic and genetic heterogeneity and be reflected in pretreatment CT images (6, 39, 40). Some findings have shown that tumor heterogeneity is a predictor of survival in patients with NSCLC (6, 41).

### Limitations and further works

There are some limitations to this study. First, the sample size was small. This made the extensive stratified analysis unfeasible, such as investigating the difference between adenocarcinoma and squamous cell carcinoma. Second, the segmentation of intra-and peritumoral regions is semi-automatic, and some features might be dependent on segmentation results. Automatic segmentation by deep learning and extraction of features from the bounding box may address this problem (42). Third, only machine learning methods are employed. Deep learning can be utilized as a powerful end-to-end solution or classifier (43–46).

## Conclusion

Non-contrast CT radiomic features from both the peri- and intratumoral regions can predict chemotherapy response in NSCLC *via* machine learning models. The 0–3 mm peritumoral region presented better performance than the peri- and intratumoral regions. The combined features from the two peritumoral regions may further improve the prediction. With the further evaluation of generalizability, the developed model and identified features may help improve the management of patients with NSCLC in precision medicine.

## Data availability statement

The datasets presented in this article are not readily available because they must be approved by the Ethics Committee of Shengjing Hospital of Chinese Medical University and the Fifth Affiliated Hospital of Guangzhou Medical University. Requests to access the datasets should be directed to Shouliang Qi, qisl@bmie.neu.edu.cn.

## Ethics statement

The studies involving human participants were reviewed and approved by the Ethics Committee of Shengjing Hospital of China Medical University and the Fifth Affiliated Hospital of Guangzhou Medical University. Written informed consent for participation was not required for this study in accordance with the national legislation and the institutional requirements.

## Author contributions

RC performed experiments and analyzed the data. SQ, YZ, and WQ proposed the idea, made discussions, and composed the manuscript together with RC. YY, XZ, and YG collected and analyzed the data. JS directed the algorithm development and analyzed the data. All authors have read and approved the final manuscript.

## Funding

This work was partly supported by the National Natural Science Foundation of China (82072008), Liaoning Natural Science Foundation (2011-YGJC-21), Key R&D Program Guidance Projects in Liaoning Province (2019JH8/10300051), and the Fundamental Research Funds for the Central Universities (N2119010, N2224001-10).

## Acknowledgments

Some subjects in Dataset 1 (n=250) have been previously used in Chang R, Qi S, Yue Y, Zhang X, Song J and Qian W (2021) Predictive Radiomic Models for the Chemotherapy Response in NonSmall-Cell Lung Cancer based on Computerized-Tomography Images. Front. Oncol. 11:646190. doi: 10.3389/fonc.2021.646190.

## Conflict of interest

The authors declare that the research was conducted in the absence of any commercial or financial relationships that could be construed as a potential conflict of interest.

## Publisher’s note

All claims expressed in this article are solely those of the authors and do not necessarily represent those of their affiliated organizations, or those of the publisher, the editors and the reviewers. Any product that may be evaluated in this article, or claim that may be made by its manufacturer, is not guaranteed or endorsed by the publisher.
