# Supplementary material for: Predicting chemotherapy response in non-small-cell lung cancer via computed tomography radiomic features: Peritumoral, intratumoral, or combined?
Source: Front Oncol. 2022 Aug 8;12:915835. doi: 10.3389/fonc.2022.915835 (PMC9393703; doi:10.3389/fonc.2022.915835)

**Supplementary Material for**

**Predicting Chemotherapy Response in Non-Small-Cell Lung Cancer via Computed Tomography Radiomic Features: Peritumoral, Intratumoral, or Combined?**

**Table S1. Parameters for CT image acquisition.**

| Parameters | Dataset 1 | Dataset 2 |
| --- | --- | --- |
| kVp (kV) | 120 | 120 |
| X-ray tube current (mean ± SD) (mA) | 217.68 ± 67.83 | 265.74 ± 56.25 |
| Slice thickness (mm) | 2.5 (*n =* 14); 3.0 (*n =* 239); 5.0 (*n =* 19) | 2.0 (*n* = 43) |
| Pixel size (mm) | 0.78 ± 0.07 | 0.84 ± 0.12 |
| CT scanner manufacturer | GE Medical (*n =* 10), Siemens (*n =* 10),  Toshiba (*n =* 12), Philips (*n =* 240) | Siemens (*n =* 43) |

kVp, kilovolts peak; SD, standard deviation.

**Fig. S1. Flowchart of patient selection.**


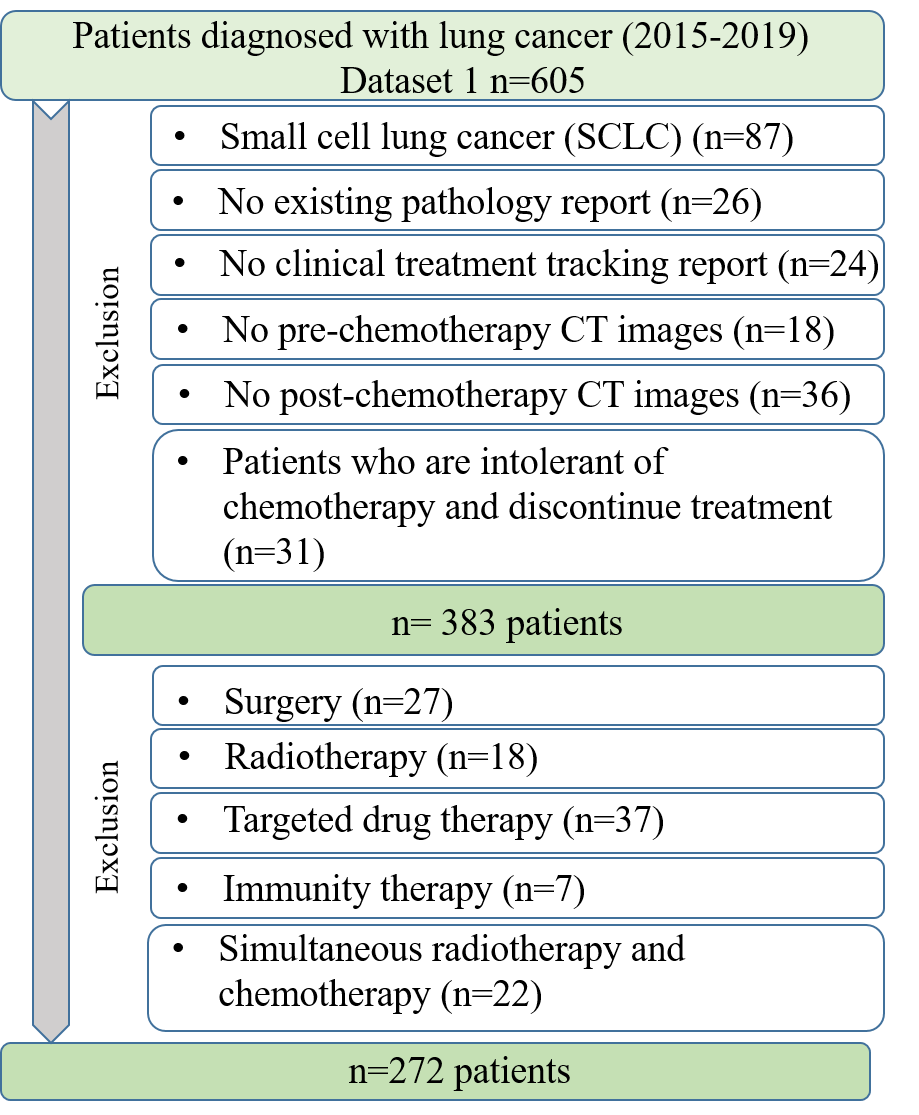

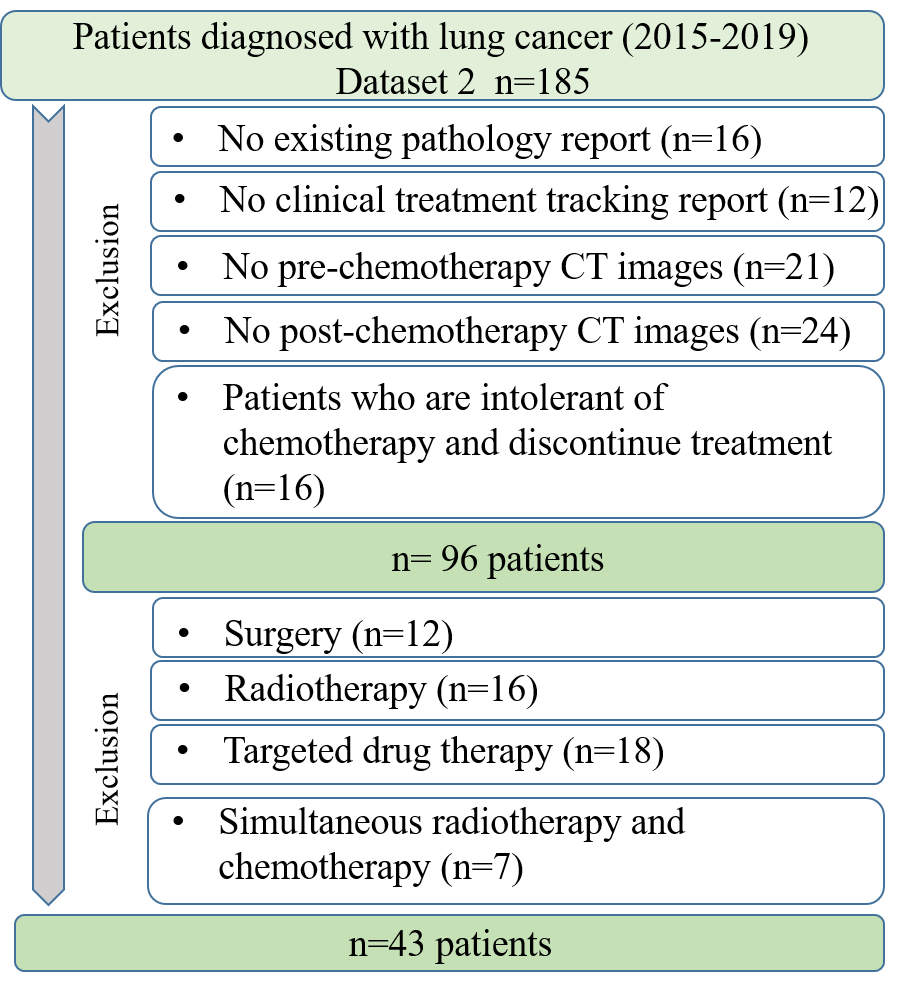


**Fig. S2. Segmentation of intra- and peritumoral regions: (a) Original CT images; (b) Segmented intratumoral region; (c) 3D view of intratumoral region; (d) Segmented peritumoral regions; (e) 3D view of peritumoral regions.**


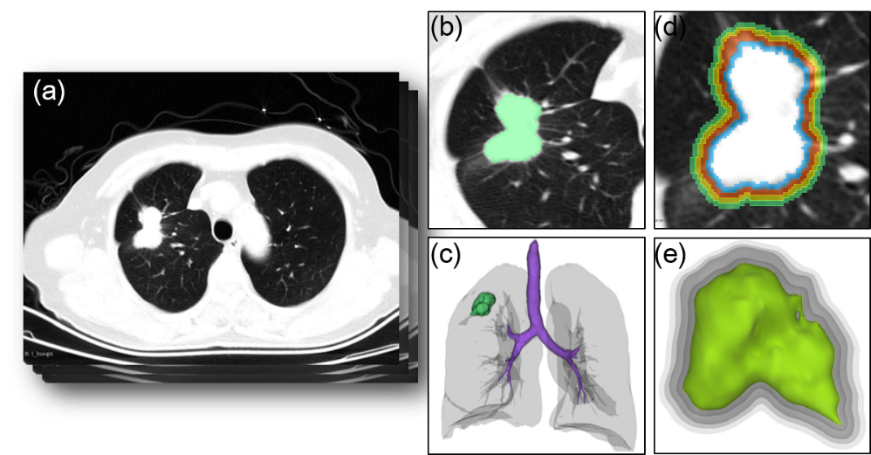


**Fig. S3. Characteristic coefficient contraction of LASSO.**

**
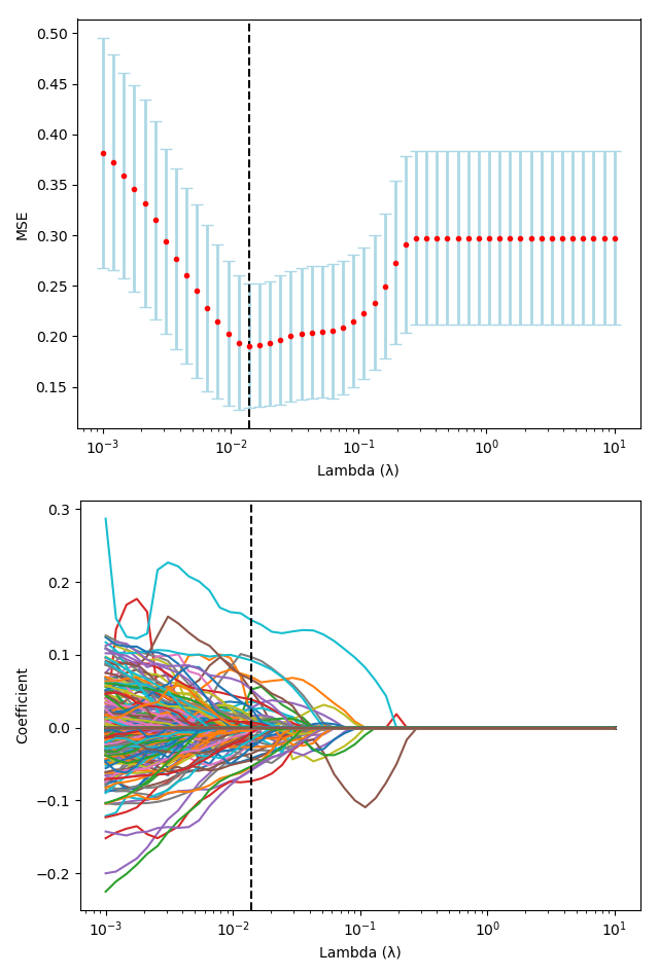
**

**Fig. S4. Comparison of models using intratumoral regions in the independent test cohort: (a) ROC curve of models; (b) Confusion matrix of the model using logistic regression.**


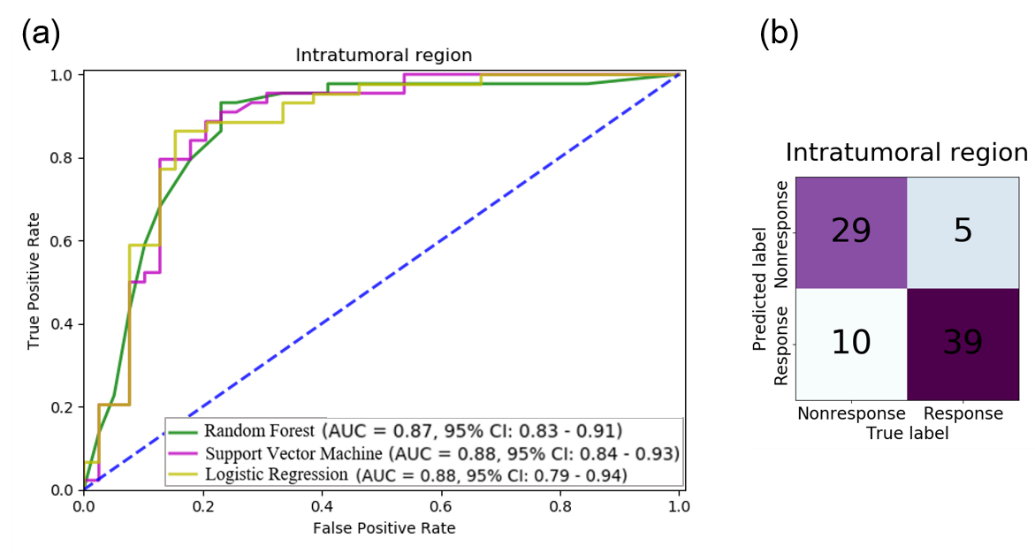


**Fig. S5. Comparison of models with different fusion methods of intra- and peritumoral region in the independent test cohort: (a) ROC curves of models using two fusion methods and three machine learning methods; (b) ROC curves of models using two fusion methods and logistic regression; (c) Confusion matrix of models using two fusion methods and logistic regression.**


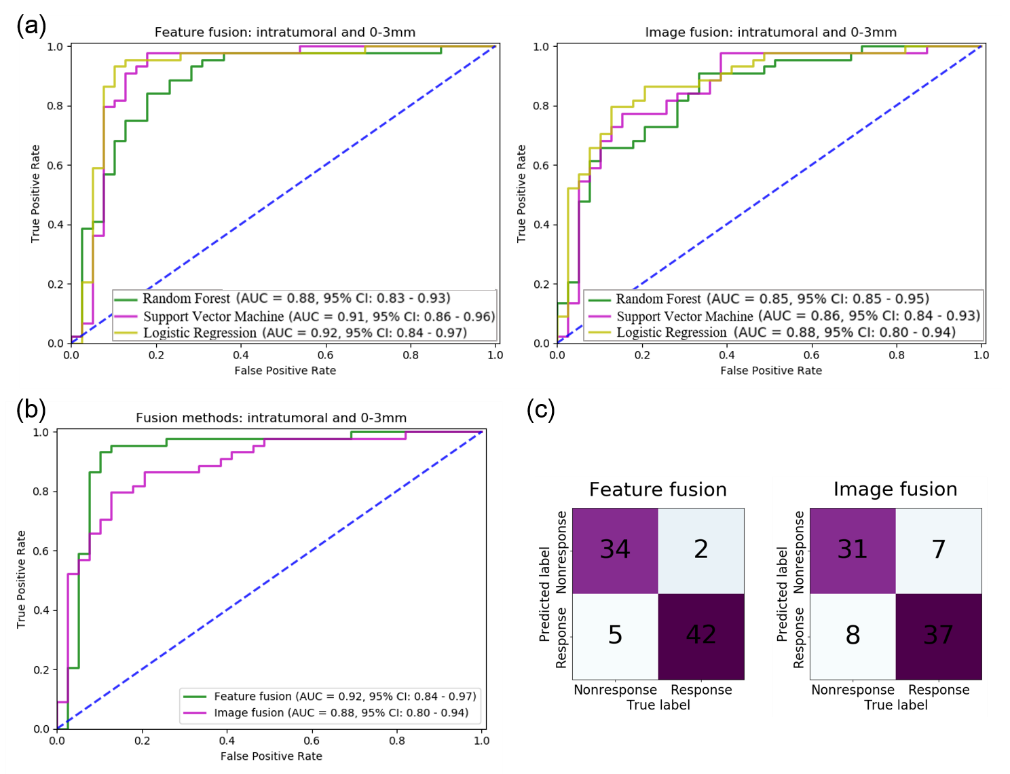


**Fig. S6. Comparison of models with clinical features and radiomics features in the independent test cohort.**


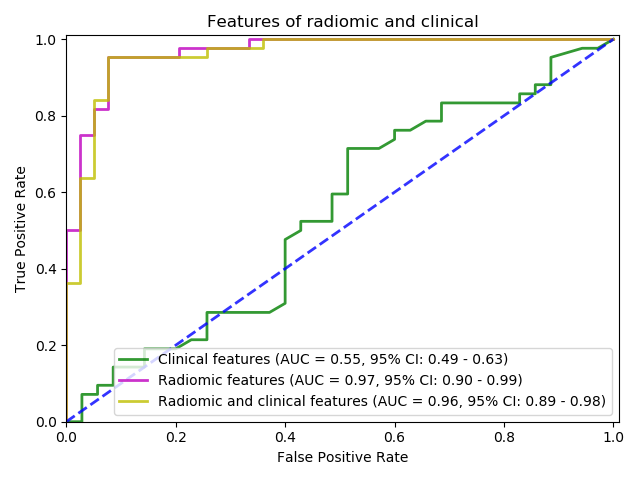

Supplement: Supplementary file 1 [file DataSheet_1.docx]
